# Serum feline pancreatic lipase immunoreactivity and trypsin‐like immunoreactivity concentrations in cats with experimentally induced chronic kidney disease

**DOI:** 10.1111/jvim.16296

**Published:** 2021-11-05

**Authors:** Panagiotis G. Xenoulis, Katerina T. Moraiti, Delmar R. Finco, Jan S. Suchodolski, Jörg M. Steiner

**Affiliations:** ^1^ Texas A&M University, College of Veterinary Medicine and Biomedical Sciences Gastrointestinal Laboratory College Station Texas USA; ^2^ Small Animal Clinic University of Thessaly Karditsa Greece; ^3^ Department of Medicine University of Thessaly Karditsa Greece; ^4^ Department of Physiology and Pharmacology University of Georgia Athens Georgia USA; ^5^ Small Animal Sciences GI Laboratory College Station Texas USA; ^6^ VSCS GI Lab College Station Texas USA

**Keywords:** azotemia, gastroenterology, pancreas, pancreatic disease, pancreatic insufficiency, pancreatic lipase, pancreatitis, renal disease, renal/urinary tract

## Abstract

**Background:**

Serum feline pancreatic lipase immunoreactivity (fPLI) and trypsin‐like immunoreactivity (fTLI) concentrations are commonly used in cats for the evaluation of pancreatic disease. The effect of kidney disease on these tests in cats are unknown.

**Objective:**

To investigate the effect of experimentally induced chronic kidney disease (CKD) on serum fPLI and fTLI concentrations.

**Animals:**

Surplus serum samples from 20 cats with CKD experimentally induced for an unrelated project and a group of healthy control cats.

**Methods:**

Serum fTLI and fPLI concentrations were compared between groups.

**Results:**

Mean (±SD) serum fTLI concentrations in 20 cats with CKD (117.8 ± 63.6 μg/L) were significantly higher than those in healthy cats (n = 32; 46.9 ± 17.5 μg/L; *P* < .0001). Serum fTLI concentrations in cats with CKD were above the upper limit of the reference interval in 13 of 20 cats (65%). Serum fPLI concentrations were not significantly different between cats with induced CKD (n = 18; 8.6 μg/L; range, 5.4‐9.9 μg/L) and healthy cats (n = 41; 7.4 μg/L; range, 5.0‐15.2 μg/L; *P* = .12). All cats with experimentally induced CKD had serum fPLI concentrations within the reference interval.

**Conclusions and Clinical Importance:**

Decreased renal function has a clinically relevant impact on serum fTLI concentrations and potentially could interfere with a diagnosis of exocrine pancreatic insufficiency (EPI). Serum fPLI concentration was not affected by experimentally induced CKD and thus serum fPLI may be used for the diagnosis of pancreatitis in cats with kidney disease. Additional studies are needed to verify these results in cats with naturally occurring CKD.

## 1 INTRODUCTION

Exocrine pancreatic diseases in cats are common in clinical practice. Pancreatitis is the most common and clinically important disease of the exocrine pancreas in this species.^1^, ^2^ Exocrine pancreatic insufficiency (EPI), although once considered rare, is the next most common disease of the exocrine pancreas in cats and is diagnosed more frequently than in the past.^3^

Although pancreatitis occurs frequently in cats, its clinical diagnosis remains challenging because of its nonspecific clinical signs, which often resemble those of other conditions that complicate or occur concurrently with pancreatitis (eg, anorexia, lethargy, weight loss, vomiting) and nonspecific clinicopathologic findings.^4^, ^5^, ^6^ Histopathology of the pancreas is considered the gold standard for the diagnosis of pancreatitis in cats, but it is uncommonly used because of its invasiveness, high cost, and certain diagnostic limitations (such as the lack of a standardized classification scheme and the possibility of missing highly localized lesions).^1^, ^7^ The exclusion of other diseases and the use of sensitive and specific serum markers and imaging modalities (such as abdominal ultrasound examination) are now considered the most useful means for a clinical diagnosis of pancreatitis in cats with compatible clinical presentation.^7^ The most useful serum marker for the diagnosis of pancreatitis in cats currently is considered the measurement of feline pancreatic‐lipase immunoreactivity (fPLI, measured as Spec fPL), which has been reported to have a sensitivity of 54% to 82% and a specificity of 67% to 100%.^6^, ^7^, ^8^, ^9^

The clinical presentation of cats with EPI is nonspecific and may differ from the presentation in dogs with EPI. Although EPI was once believed to be extremely rare in cats, since the development of feline trypsin‐like immunoreactivity (fTLI) assay, it has been shown that EPI does occur in cats with considerable frequency.^3^, ^10^, ^11^ Serum fTLI concentrations now are considered the test of choice for the diagnosis of EPI in cats, with sensitivity and a specificity of 85% to 100%.^3^, ^10^, ^11^

Both serum fPLI and fTLI concentrations may be affected by extrapancreatic conditions, which can lead to decreased specificity of these assays. Azotemia is a common clinicopathologic finding in cats with pancreatitis. Some affected cats have prerenal azotemia, which may be the result of dehydration caused by vomiting, diarrhea, decreased water intake or some combination of these.^12^, ^13^ Renal azotemia also has been commonly reported in association with pancreatitis in cats, reflecting decreased glomerular filtration rate (GFR) as a result of complicating or concurrent acute or chronic kidney disease (CKD).^12^, ^13^, ^14^ However, the association between pancreatitis and kidney disease in cats remains obscure. Renal azotemia may reflect concurrent CKD especially in cats of older age, but pancreatitis may lead to acute kidney injury by hypovolemia, ischemia, and inflammation. Decreased GFR is known to affect several serum biomarkers and the same may be true for fTLI and fPLI concentrations. To date, the effect of CKD on serum fTLI and fPLI concentrations has not been critically evaluated.

Our aim was to evaluate serum fTLI and fPLI concentrations in cats with decreased GFR associated with experimentally induced CKD.

## 2 MATERIALS AND METHODS

### 2.1 Serum from cats with CKD

Surplus serum from 20 young (10‐11 months old), female, purpose‐bred cats that had been part of a previous unrelated study^15^ was used. These cats had been deemed healthy based on physical examination, CBC, serum biochemical analysis, and urinalysis. Renal mass was decreased surgically in these cats as previously described^16^ and the cats were allowed to recover from surgery for 2 months. All cats were monitored daily for development of clinical signs, were followed up for 12 months, and blood samples were collected at 2‐month intervals.

Only surplus serum samples (stored at −80°C) from these cats were used in the present study and no animal research was conducted for the purpose of the present study. Although samples were collected at different timepoints after the induction of CKD as part of the original study protocol, only samples from a single time point for each cat were available for use in the present study. Serum fTLI concentrations were determined 3 years after sample collection, whereas serum fPLI and Spec fPL concentrations were measured 9 years after sample collection.

### 2.2 Healthy control cats

A group of healthy cats was used as controls. These cats were considered healthy based on history, physical examination, and routine blood testing. The age of the cats ranged between 1 and 5 years. The cats were owned by students and staff of Texas A&M University and the study protocol was reviewed and approved by the Texas A&M University Clinical Research Review Committee (CRRC# 2008‐37, approved November 5, 2008). Serum fTLI concentrations were measured in 32 healthy cats, and serum fPLI concentrations were measured in a different group of 41 healthy cats.

### 2.3 Assays

All measurements were made as a batch analysis for all samples within the same assay run. Serum creatinine concentrations were measured using an automated serum chemistry analyzer. Serum fTLI concentrations were measured using an analytically validated radioimmunoassay (RIA) with a reference interval of 12 to 82 μg/L, with concentrations >100 μg/L considered indicative of pancreatitis.^10^ Serum fPLI concentrations were measured using an analytically validated RIA as described previously.^17^ The reference interval of the assay had been changed on multiple occasions because of changes in antiserum batches used, and at the time of the study it was 4.1 to 12.9 μg/L. Serum fPLI concentrations also were measured using the Spec fPL assay (Idexx Laboratories, Westbrook, ME) according to the manufacturer's instructions.^18^ The reference interval of the assay is <3.5 μg/L and concentrations >5.3 μg/L are considered consistent with pancreatitis, and concentrations between 3.5 and 5.3 μg/L are considered equivocal.^19^ All assays were performed at the Gastrointestinal Laboratory at Texas A&M University.

### 2.4 Stability of serum fTLI and fPLI concentrations

Feline pancreatic lipase is considered highly stable when stored in −80°C.^19^ To further test the stability of feline pancreatic lipase in serum samples stored at −80°C, 9 unrelated samples were tested before and after 44 months of storage at −80°C. Feline TLI is stable in serum for several days at room temperature and it is considered to be stable for years when stored at −80°C.^20^

### 2.5 Statistical analyses

All statistical analyses were performed using commercial statistical software (Prism 7, GraphPad, San Diego, CA). Data were tested for normality using the Kolmogorov‐Smirnov or the Shapiro‐Wilk test. Normally distributed data were compared between groups using Student's *t* tests, whereas non normally distributed data were compared between groups using Mann‐Whitney tests. Correlation was assessed with Pearson *r* and Spearman *r*. Significance was set at *P* < .05.

## 3 RESULTS

Serum creatinine, fTLI, and fPLI concentrations in cats with experimentally induced CKD are shown in Table S1.

### 3.1 Serum creatinine concentrations

Surplus serum was available for 20 cats with experimentally induced CKD. Serum creatinine concentrations were measured in all cats and all cats had serum creatinine concentrations above the upper limit of the reference (reference interval, ≤1.5 mg/dL; median, 2.9 mg/dL; range, 2.2‐5.4 mg/dL). These cats would be classified as stage I, II, III, and IV according to the International Renal Interest Society (IRIS) guidelines. Specifically, 1 cat was in IRIS stage I, 10 cats were in stage II, 8 cats in stage III, and 1 cat was in stage IV.

### 3.2 Serum fTLI concentrations

**FIGURE 1:**
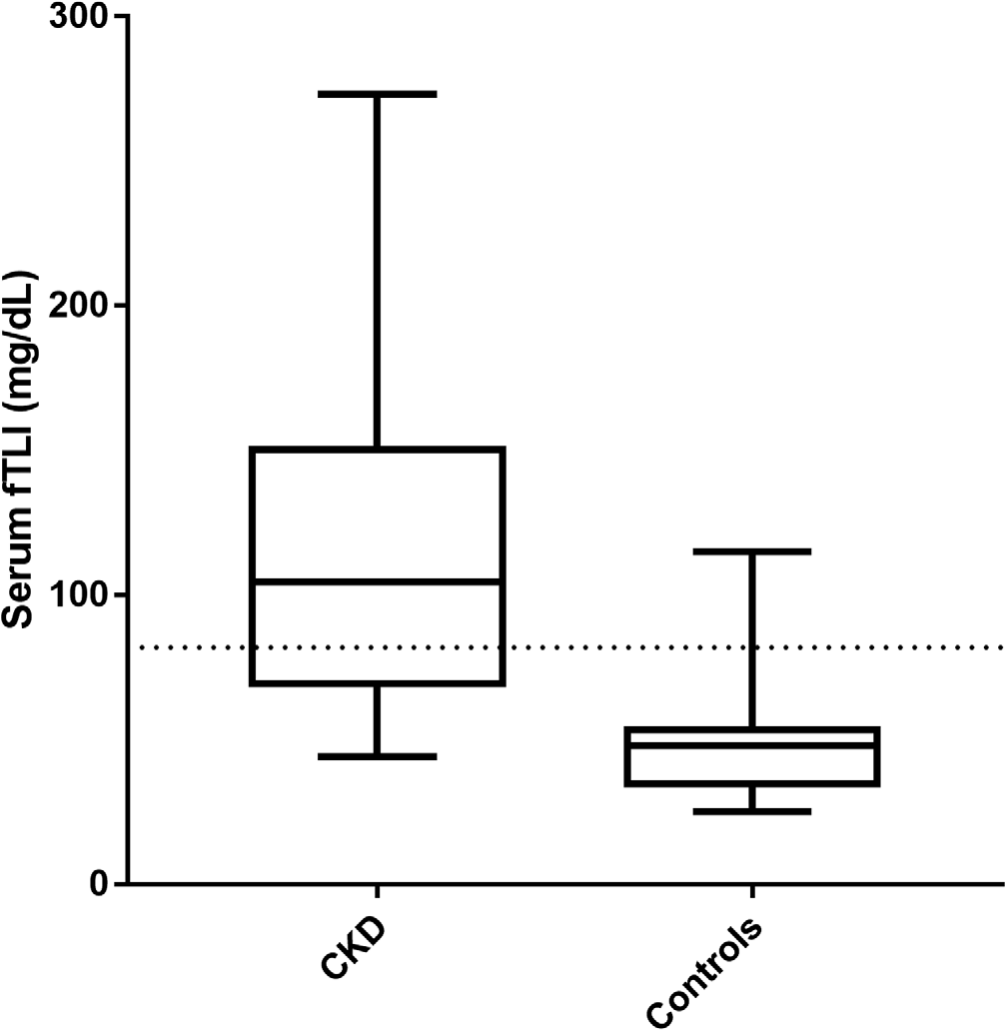
Boxplots of serum fTLI concentrations in cats with CKD and healthy control cats. The lines in the rectangles represent the median for each group. The upper and lower limits of the rectangles correspond to the 25th and 75th percentile of each group, respectively. The blue dashed line represents the upper limit of the reference interval. In 13 of 20 (65%) cats with CKD serum fTLI concentration was above the upper limit of the reference interval (82 μg/L)

**FIGURE 2:**
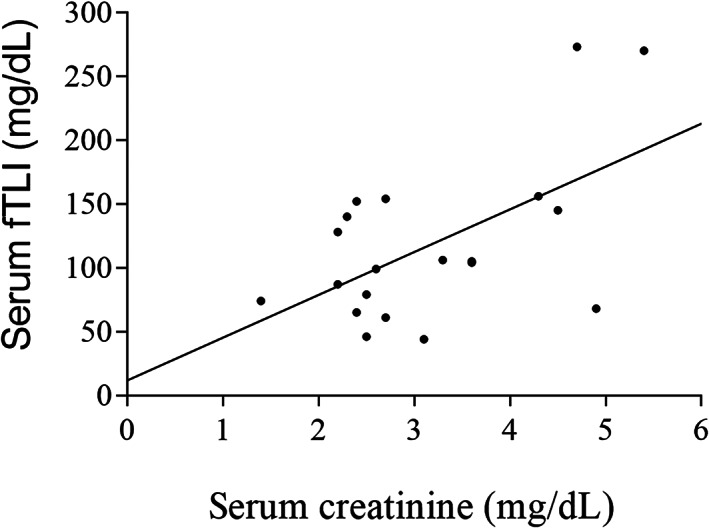
Scatterplot of serum fTLI vs serum creatinine. There was a statistically significant moderate correlation between serum fTLI and creatinine concentrations in cats with CKD (n = 20; Pearson *r* = 57; *P* = .008)

Serum fTLI concentrations were measured in all 20 cats with CKD for which serum was available. Serum fTLI concentrations in cats with CKD (median, 104.5 μg/L; range, 44‐273 μg/L) were significantly higher than those of clinically healthy cats (n = 32; median, 47.9 μg/L; range, 25‐115 μg/L; *P* < .0001; Figure 1). Serum fTLI concentrations in cats with CKD were above the upper limit of the reference interval (82 μg/L) in significantly more cats (13 of 20 cats, 65%) compared to healthy control cats (2 of 32 cats; 6%; *P* < .0001). A statistically significant moderate correlation was found between serum fTLI and creatinine concentrations in cats with CKD (Pearson *r* = 57; *P* = .008; Figure 2).

### 3.3 Serum fPLI and fPL concentrations

**FIGURE 3:**
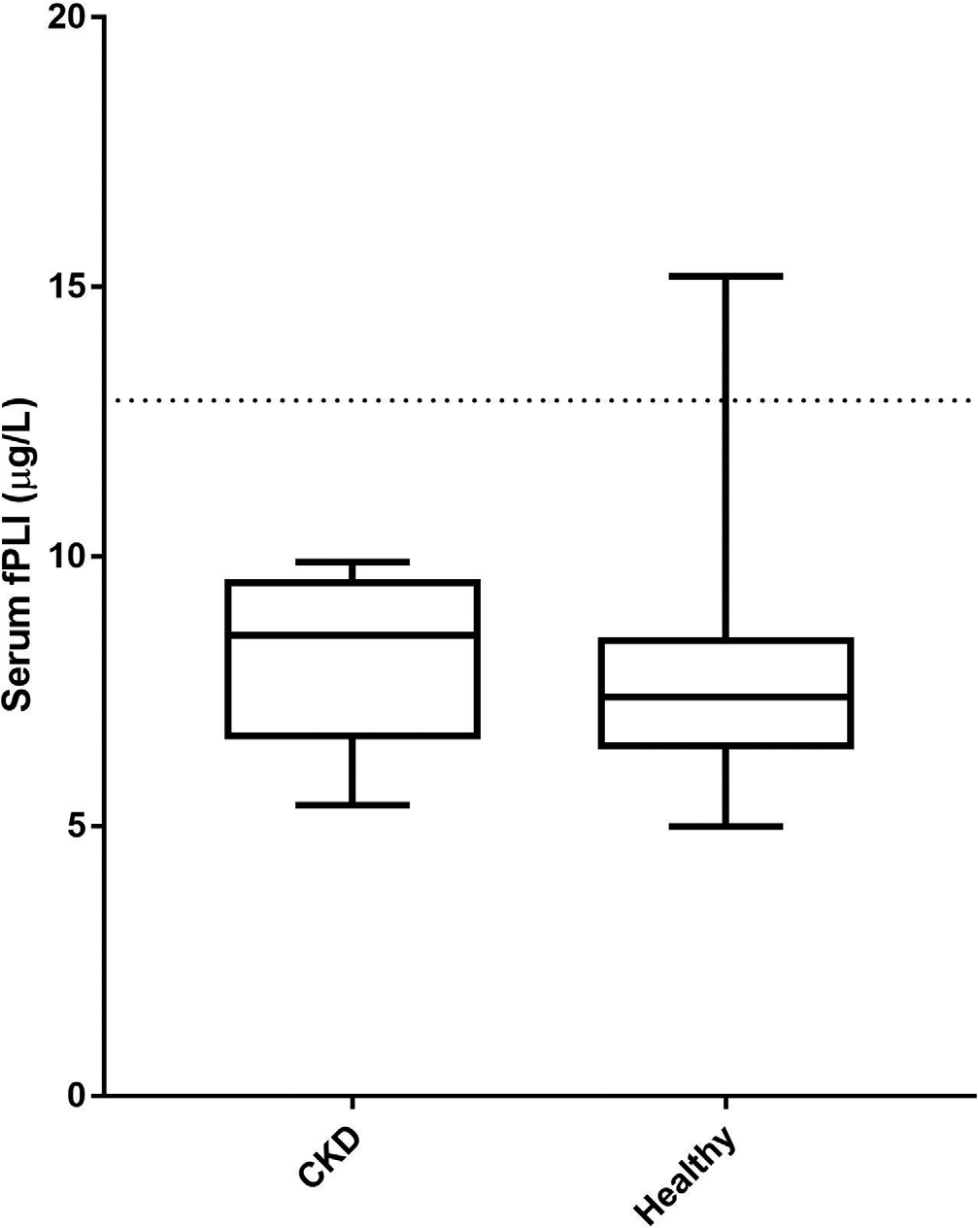
Boxplots of serum fPLI concentrations as measured by an in‐house radioimmunoassay in cats with CKD (n = 20) and healthy control cats (n = 31). The lines in the rectangles represent the median for each group. The upper and lower limits of the rectangles correspond to the 25th and 75th percentile of each group, respectively. The blue dashed line represents the upper limit of the reference interval. Overall, serum fPLI concentrations were not significantly different between cats with induced CKD (median, 8.6 μg/L; range, 5.4‐9.9 μg/L) and healthy cats (median, 7.4 μg/L; range, 5.0‐15.2 μg/L; *P* = .12). All 18 cats with CKD had serum fPLI concentrations within the reference interval (4.1‐12.9 μg/L)

**FIGURE 4:**
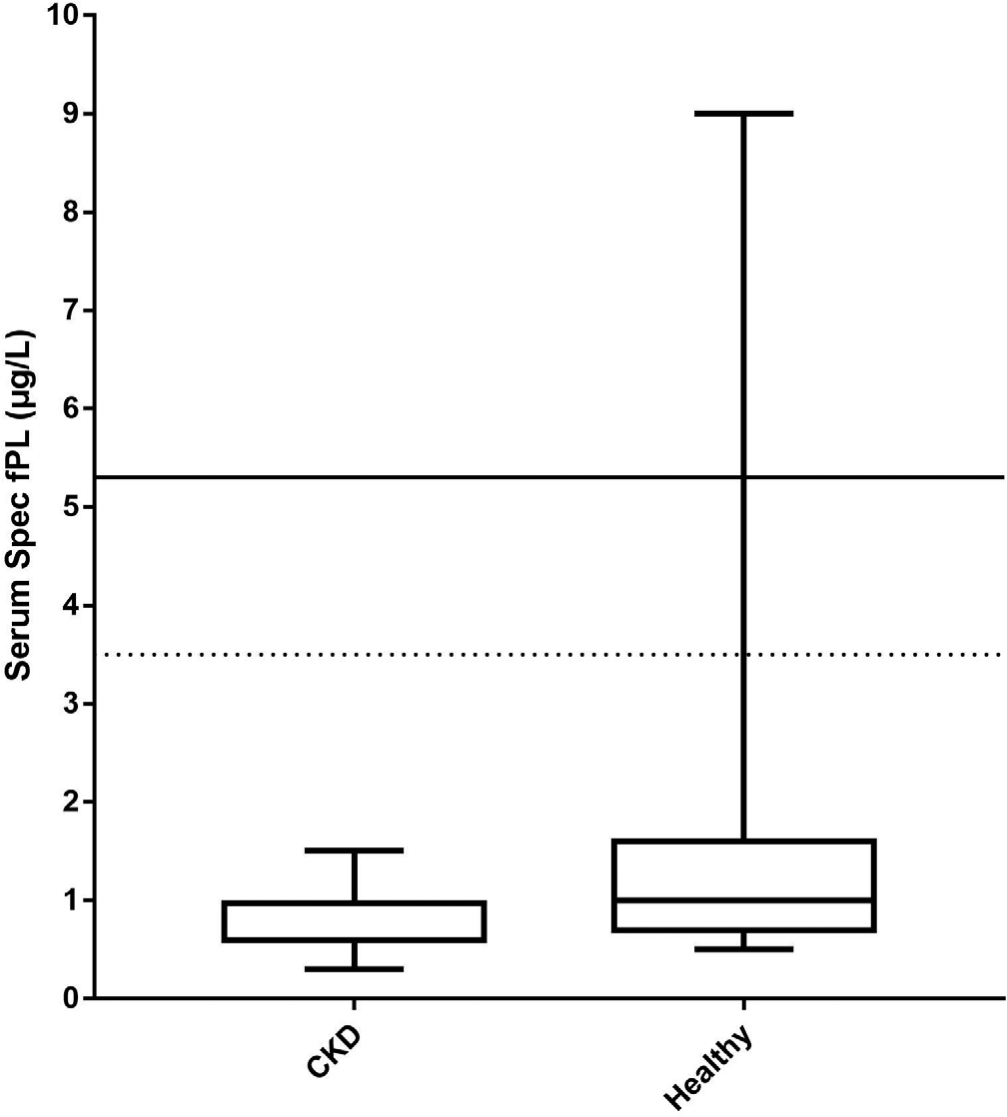
Boxplot of serum fPLI concentrations as measured by the Spec fPL assay in cats with CKD (n = 20) and healthy control cats (n = 41). The lines in the rectangles represent the median for each group. The upper and lower limits of the rectangles correspond to the 25th and 75th percentile of each group, respectively. The blue dashed line represents the upper limit of the reference interval. All 16 cats with CKD had serum fPLI concentrations within the reference interval (0‐3.5 μg/L) for this assay

**FIGURE 5:**
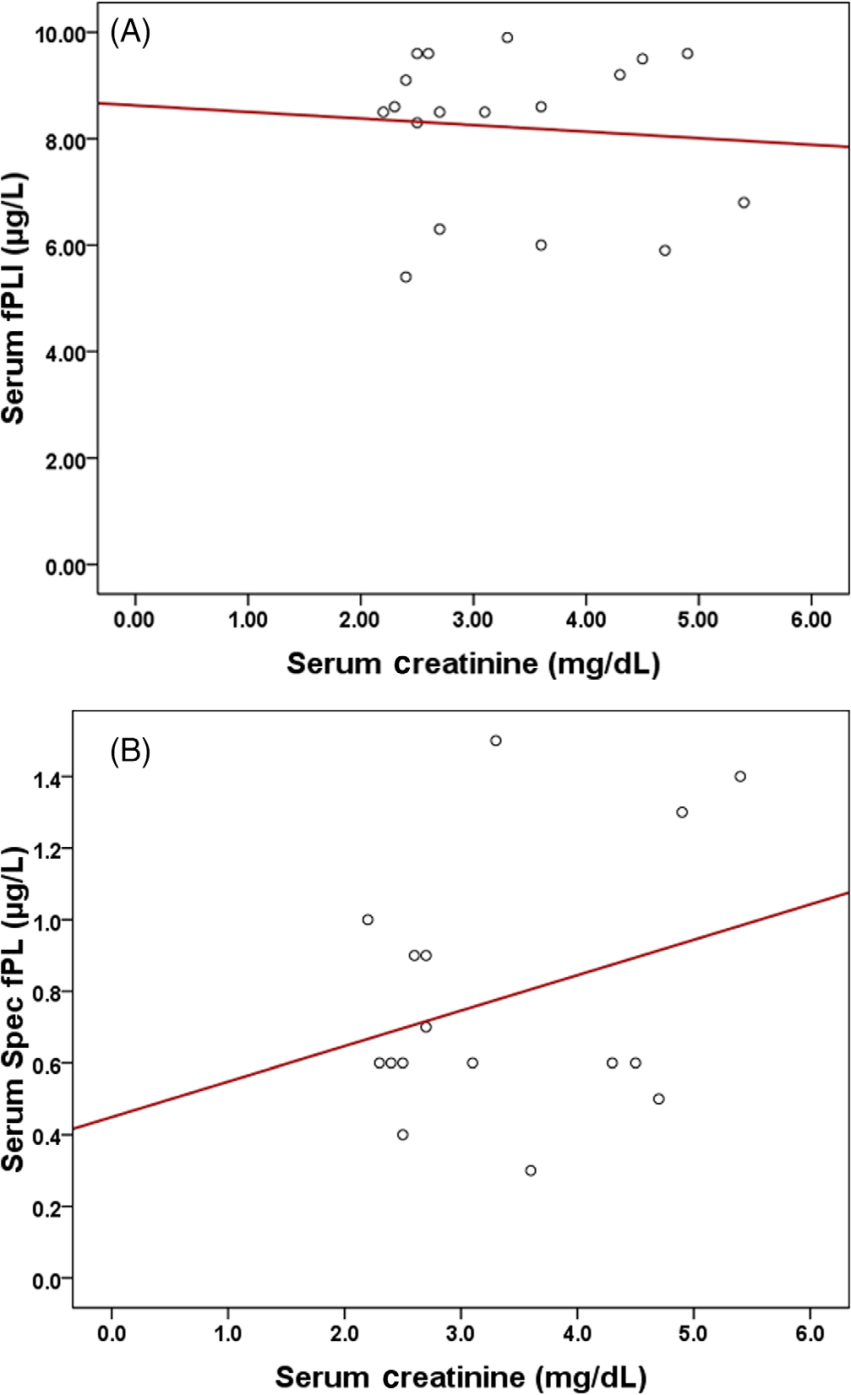
(A, B) Scatterplots of serum fPLI concentrations as measured by an in‐house RIA (A) and by the Spec fPL assay (B) vs serum creatinine. There was no significant correlation between serum creatinine concentration and fPLI concentrations by either the in‐house RIA (Spearman *r* = .037; *P* = .88) or the Spec fPL assay (Spearman *r* = .127; *P* = .64)

Serum fPLI concentrations were measured in 18 cats with CKD using the in‐house RIA and in 16 cats with CKD using the Spec fPL assay. Serum fPLI concentrations as measured by the RIA were not significantly different between cats with experimentally induced CKD (median, 8.6 μg/L; range, 5.4‐9.9 μg/L) and healthy cats (n = 41; median, 7.4 μg/L; range, 5.0‐15.2 μg/L; *P* = .12; Figure 3). Serum Spec fPL concentrations were significantly lower in cats with experimentally induced CKD (median, 0.6 μg/L; range, 0.3‐1.5 μg/L) compared to healthy cats (n = 41; median, 1 μg/L; range, 0.5‐9 μg/L; *P* = .01; Figure 4). All cats with experimentally induced CKD had serum fPLI concentrations within the reference interval of the in‐house RIA and the Spec fPL assay. In the control group, a single cat had serum fPLI and Spec fPL concentration in the diagnostic range for pancreatitis. No difference in the results was found after excluding this cat from the analysis. No significant correlation was found between serum creatinine concentration and serum fPLI concentrations either when measured by the in‐house RIA (Spearman *r* = .037; *P* = .88; Figure 5A) or the Spec fPL assay (Spearman *r* = .127; *P* = .64; Figure 5B).

### 3.4 Stability of serum fPLI concentrations

**FIGURE 6:**
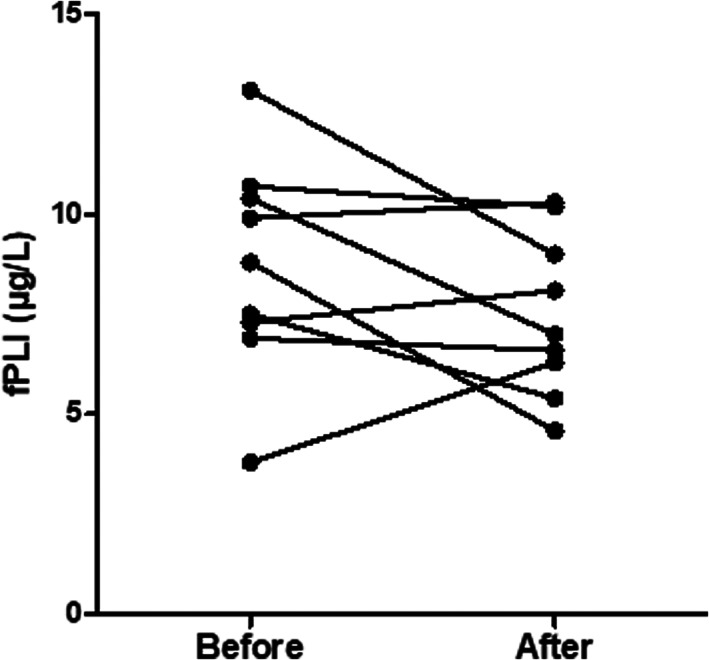
Scatterplot of serum fPLI concentrations as measured by an in‐house RIA before and after 44 months of storage at −80°C. There was no statistically significant difference of the mean serum fPLI concentrations for the 9 serum samples before (8.7 μg/L) and after 44 months of storage at −80°C (7.5 μg/L; *P* = .16; 95% confidence interval = −0.6 to 3.0)

No statistically significant difference was found in serum fPLI concentrations as measured by the in‐house RIA before and 44 months after storage at −80°C (8.7 μg/L vs 7.5 μg/L; *P* = .16; 95% confidence interval [CI], 0.6‐3.0; Figure 6).

## 4 DISCUSSION

Our results show that serum fTLI concentrations were significantly affected by and positively correlated with serum creatinine concentration in this model of experimentally induced CKD in cats. In contrast, experimentally induced CKD in these cats did not cause a significant increase in serum fPLI concentrations, either when measured by the in‐house RIA or by the Spec fPL assay. Therefore, decreased renal excretion during CKD, as documented by increases in serum creatinine concentration, significantly affected serum fTLI but not fPLI concentrations in this group of cats.

The spectrum of severity of kidney disease in the cats in our study ranged from stage II to stage IV based on IRIS classification, therefore representing a broad range of disease severity. This experimental model of CKD, however, may not accurately reflect spontaneous CKD, and differences in the metabolism and excretion of these biomarkers may occur in cats with naturally occurring kidney disease.

Renal handling of biological substances circulating in the blood is affected by size and charge (ie, factors affecting glomerular filtration), and if filtered, also by reabsorption (renal tubular handling) of these substances. In addition, other physiologic or pathophysiologic mechanisms, such as protein binding, reticuloendothelial clearance, and alterations in glomerular permselectivity might affect serum concentrations of biomarkers.

Trypsin has a relatively low molecular weight (approximately 25 kDa), is positively charged and therefore is considered to have a relatively high filtration rate. Therefore, a decrease in glomerular filtration as a result of CKD would be expected to lead to decreased filtration of trypsin and an increase in serum concentration. In our study, serum fTLI concentrations were significantly higher in cats with experimentally induced CKD compared to healthy control cats. In addition, serum fTLI concentrations were above the reference interval and the cut‐off for pancreatitis in more than half of the cats. These findings suggest that decreased clearance of trypsinogen occurs in cats with CKD. Clinically, these findings suggest that serum fTLI concentrations may be falsely increased in cats with decreased renal function. Thus, clinicians should be cautious when using serum fTLI concentrations in cats with impaired renal function to diagnose EPI, because decreased renal function in these animals potentially could falsely increase serum fTLI concentrations into or above the reference interval and interfere with a diagnosis of EPI. However, to date, this situation has not been described in cats with suspected EPI and CKD.

Our findings on serum fTLI concentrations are in contrast with a recent study in dogs. In that study, serum canine TLI concentrations were not affected by decreased renal excretion in dogs with induced acute kidney injury, but the number of dogs in that study was too small (5 dogs) and significant associations might have been missed because of type II statistical error.^21^ In fact, correlation between serum canine TLI and creatinine concentrations approached but did not reach statistical significance (*P* = .06). Additionally, species differences or differences in experimental models might have played a role in these conflicting results. No studies have evaluated serum fTLI concentrations in cats with spontaneous renal disease.

Feline pancreatic lipase has a relatively high molecular weight (approximately 52.5 kDa) and a negative charge, and therefore, it is considered to have relatively low filtration. Therefore, a decrease in glomerular filtration as a result of CKD would not be expected to have a clinically relevant impact on serum fPLI concentration. Indeed, serum fPLI concentrations, measured by an in‐house RIA or by the Spec fPL assay, were not significantly different between cats with experimentally induced CKD and healthy control cats. The fact that serum pancreatic lipase concentrations are not affected by impaired renal function also is illustrated by the fact that there was no significant correlation between serum creatinine concentrations and fPLI concentrations.

Our results mirror those of previous studies in dogs where serum canine PLI concentrations were not affected by decreased renal function in dogs with induced acute kidney injury.^22^ In another study, dogs with experimentally induced renal failure had significant, but clinically unimportant, increases in serum canine PLI concentrations, and they remained below the cut‐off for pancreatitis in all dogs.^21^

Our results contradict those of the only other study investigating the association between serum feline pancreatic lipase concentrations and kidney disease in cats. In that study,^23^ the effect of azotemia and naturally occurring kidney disease (as defined by increased serum creatinine concentrations and urine specific gravity <1.035) on serum feline pancreatic lipase was investigated in 2189 samples. Serum Spec fPL concentration was increased in 26% of cats overall, whereas 17% of cats had serum Spec fPL concentrations in the diagnostic range for pancreatitis. In cats with CKD, serum Spec fPL concentration was increased in 50% of cats, whereas 36% of cats had serum Spec fPL concentrations diagnostic for pancreatitis. Measures of statistical significance (ie, odds ratio and p value) were not mentioned for these categorical variables. No correlation was found between serum Spec fPL and creatinine concentrations. Based on the above evidence, it was concluded that serum Spec fPL concentration is affected by naturally occurring kidney disease. However, that study had several limitations. First, no history, clinical examination findings, imaging findings or histopathological findings were available for any of those cats and is therefore unknown how many of those cats might have had concurrent pancreatitis. Because of the common coexistence of pancreatitis and azotemia (renal and prerenal), many of the cats with azotemia and increased serum Spec fPL concentrations in that study would be assumed to also have had pancreatitis. Also, a previous study has described the concurrent occurrence of nephritis and pancreatitis in cats.^12^ Therefore, no clear conclusions can be drawn from the previous study.

Renal handling of lipase (and pancreatic enzymes in general) is not fully understood.^24^, ^25^, ^26^ Some studies show an increase in serum lipase concentration in patients with CKD. Differences in the results between studies in humans and our study potentially can be explained. First, human pancreatic lipase has lower molecular weight compared to that of the cat (46 vs 52.5 kDa).^24^, ^25^, ^26^ More importantly, measurement of human lipase is based on activity assays, which do not specifically measure pancreatic lipase.^24^, ^25^, ^26^ Lipases originate from a variety of tissues (eg, pancreas, liver, stomach endothelium) and increases in serum lipase based on activity assays are known to have low specificity, especially in cats.

Our study had several limitations. First, the samples used were from a previous study, meaning that the study design was predetermined and could not be optimized for the purpose of our study. Ideally, additional serum samples would have been collected and analyzed before the induction of kidney disease and also at different timepoints during the development of the disease, but such samples were not available. Second, there might be concerns regarding stability of serum trypsinogen and pancreatic lipase. However, these molecules have been shown to have unusual resilience even at room temperature compared to other proteins that deteriorate much faster.^20^, ^22^, ^27^ In addition, in our stability study, serum fPLI concentrations were unaffected after storage for 44 months at −80°C. Samples from cats with CKD were analyzed for serum fPLI and Spec fPL concentrations almost 9 years after collection, which is longer than the 44 months of our stability study. However, in the stability study, there was no evidence of decreases in serum fPLI or Spec fPL concentrations over an almost 4‐year period, and if there was degradation of pancreatic lipase it would be expected to happen gradually over years, and so some evidence of reduction in serum fPLI and Spec fPL concentrations should have been detected in our stability study. Finally, urinalysis was not available for the healthy control cats in our study, and therefore, early‐stage CKD might have been present in some of these cats and potentially could have been identified based on subnormal urine specific gravity.

## 5 CONCLUSIONS AND CLINICAL IMPORTANCE

Our results suggest that experimentally induced CKD in cats leads to significantly increased serum fTLI concentrations. Therefore, evaluation of serum fTLI concentrations in azotemic cats with impaired kidney function may interfere with a diagnosis of EPI in cats with CKD. In contrast, serum fPLI concentrations were not affected by experimentally induced CKD either when measured using an in‐house RIA or the Spec fPL assay. Thus, serum fPLI concentrations may be used for the diagnosis of pancreatitis in cats with impaired renal function. Increases in serum fPLI concentrations in azotemic cats (with the severity of kidney dysfunction achieved in this study) are likely specific for pancreatitis. Additional studies in cats with naturally occurring CKD or with acute kidney injury are warranted to verify these results for these groups of patients.

## CONFLICT OF INTEREST DECLARATION

Authors declare no conflict of interest.

## OFF‐LABEL ANTIMICROBIAL DECLARATION

Authors declare no off‐label use of antimicrobials.

## INSTITUTIONAL ANIMAL CARE AND USE COMMITTEE (IACUC) OR OTHER APPROVAL DECLARATION

Authors declare no IACUC or other approval was needed.

## HUMAN ETHICS APPROVAL DECLARATION

Authors declare human ethics approval was not needed for this study.

## Supporting information


**Table S1** Serum creatinine, fTLI, and fPLI concentrations as measured by an in‐house RIA and by the Spec fPL assay in cats with experimentally induced chronic kidney disease.
