# Supplementary material for: Serum feline pancreatic lipase immunoreactivity and trypsin‐like immunoreactivity concentrations in cats with experimentally induced chronic kidney disease
Source: J Vet Intern Med. 2021 Nov 5;35(6):2821–7. doi: 10.1111/jvim.16296 (PMC8692220; doi:10.1111/jvim.16296)
Supplement: Supplementary file 1 — Table S1 Serum creatinine, fTLI, and fPLI concentrations as measured by an in‐house RIA and by the Spec fPL assay in cats with experimentally induced chronic kidney disease. [file JVIM-35-2821-s001.pdf]

ID Number	**CREA (mg/dL)**	**IRIS stage**	**fTLI (µg/L)**	**fPLI - RIA (µg/L)**	**fPLI - Spec fPL (µg/L)**
**1**	1.4	I	74	N/A	N/A
**2**	4.9	III	68	9.6	1.3
**3**	4.3	III	156	9.2	0.6
**4**	3.3	III	106	9.9	1.5
**5**	3.6	III	105	6.0	N/A
**6**	2.5	II	46	8.3	0.4
**7**	2.3	II	140	8.6	0.6
**8**	4.5	III	145	9.5	0.6
**9**	2.2	II	128	8.5	1.0
**10**	3.1	III	44	8.5	0.6
**11**	2.7	II	61	6.3	0.7
**12**	2.5	II	79	9.6	0.6
**13**	2.4	II	65	9.1	0.6
**14**	3.6	III	104	8.6	0.3
**15**	5.4	IV	270	6.8	1.4
**16**	4.7	III	275	5.9	0.5
**17**	2.7	II	154	8.5	0.9
**18**	2.6	II	99	9.6	0.9
**19**	2.4	II	152	5.4	N/A
**20**	2.2	II	87	N/A	N/A
**Mean**	3.2		118	8.2	0.7
**Median**	2.7		105	8.6	0.6

Supplemental Table 1. Serum creatinine, fTLI, and fPLI concentrations as measured by an inhouse RIA and by the Spec fPL assay in cats with experimentally induced chronic kidney disease.
